# Allergic Rhinitis and Treatment Modalities: A Review of Literature

**DOI:** 10.7759/cureus.28501

**Published:** 2022-08-28

**Authors:** Kapil Sharma, Shivani Akre, Swarupa Chakole, Mayur B Wanjari

**Affiliations:** 1 Medicine, Jawaharlal Nehru Medical College, Datta Meghe Institute of Medical Sciences, Wardha, IND; 2 Community Medicine, Jawaharlal Nehru Medical College, Datta Meghe Institute of Medical Sciences, Wardha, IND; 3 Research, Jawaharlal Nehru Medical College, Datta Meghe Institute of Medical Sciences, Wardha, IND

**Keywords:** comorbidities, histamine, mast cells, allergens, rhinitis

## Abstract

Allergic rhinitis is a highly prevalent disease affecting the quality of life of millions of patients. Immunotherapy, medication, and allergen avoidance are all part of the treatment for allergic rhinitis. Allergic rhinitis causes an increase in inflammation throughout the body. As a result, asthma, chronic hyperplastic eosinophilic sinusitis, nasal polyposis, and serous otitis media are all associated with allergic rhinitis. Treatment that is effective should target systemic inflammation and its underlying causes. It has a negative impact on work productivity and academic achievement in both children and adults. Understanding the pathophysiology of allergic rhinitis, how it relates to its comorbid disorders, and how different therapy choices affect the pathophysiology of both allergic rhinitis and its related comorbidities are essential for providing effective treatment. As the quality of air around us is changing, there is an increased chance of allergies. Along with nasal and ocular symptoms that are directly linked to the allergic process, these symptoms' interference with sleep results in daytime tiredness and a decreased quality of life. In this paper, we look at pathogenesis, causes, signs, symptoms, and treatment modalities in patients with allergic rhinitis.

## Introduction and background

The worldwide majority of people are afflicted by allergic rhinitis (AR), a chronic illness [1]. From a management point of view, it is crucial to detect patients suffering from a severe form of AR. It is IgE-mediated early and late-phase hypersensitivity responses [1,2]. Because of the non-life-threatening nature of symptoms, AR in the past has been considered a trivial disease. Congestion, itching, rhinorrhea, and sneezing are some of the nasal symptoms of AR, in addition to ocular symptoms like itchy, watery eyes, and redness. It has been demonstrated that AR has a detrimental effect on productivity and lifestyle quality, including the development of emotional challenges and a decline in sleep, social interaction, daily activities, and work and academic performance. In fact, it has been demonstrated that the global loss of job productivity caused by AR is noticeably bigger than that caused by high blood pressure and diabetes. Comorbidities of the upper and lower airways, such as rhinosinusitis and asthma, can worsen AR and may be a factor in the observed impact on productivity and quality of life [2]. Nearly 50% of participants in a study of more than 500 people from Europe said that AR was affecting their ability to sleep, decreasing their quality of life [3]. The itching in the mouth and nose, the runny or stuffy nose, and other head symptoms were the AR symptoms that were most commonly reported to interfere with sleep [3].

## Review

Pathogenesis and causes

**Figure 1:**
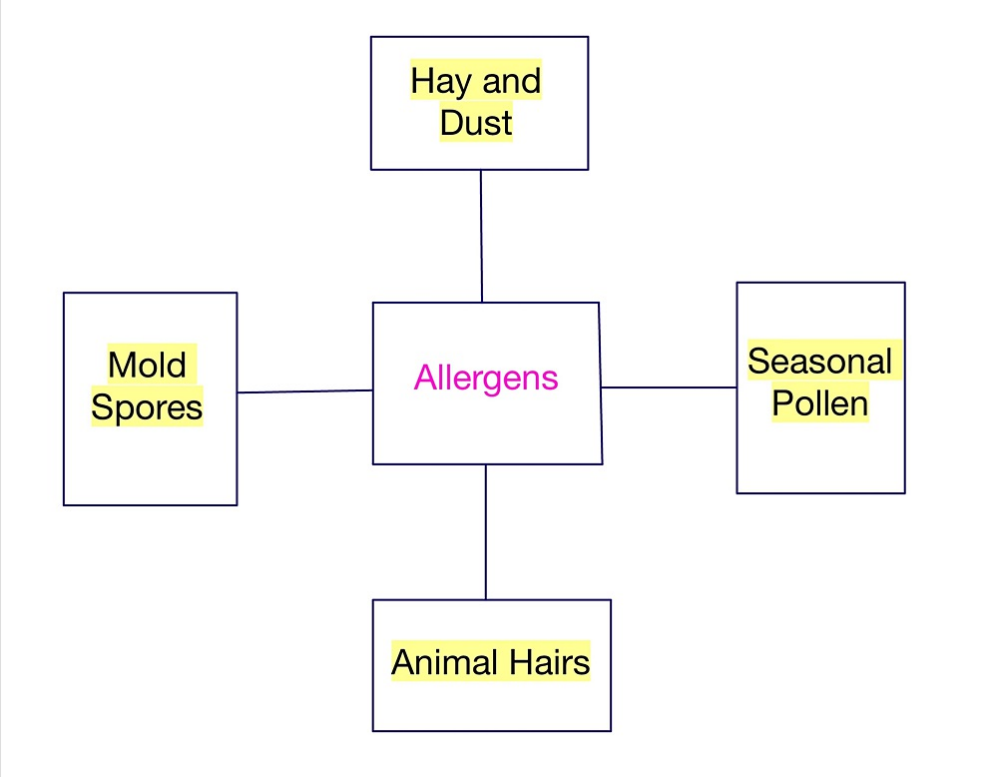
Different types of allergens causing allergic rhinitis

Rhinitis can be brought on by triggers that are allergic, non-allergic, or both (mixed rhinitis). Thus, depending on the kind of rhinitis, different underlying mechanisms result in different types of nasal symptoms. Lower respiratory tract infections, nasal polyposis (NP), sinusitis, otitis media, as well as dental malocclusion are just a few of the concomitant disorders that AR is linked to [4]. The cause of allergic rhinitis is an allergen (Figure 1).

Mechanism of inflammation

Accordingly, the fundamental process causing nasal symptoms differs per rhinitis type. Only those with a hereditary susceptibility to allergies have allergic rhinitis. Despite the fact that everyone is continually exposed to environmental allergens, only persons who have the natural capacity to become sensitized experience symptoms. Repeated exposure to aeroallergens in these sensitive people triggers B cell activation and plasma cell maturation, which results in the production of certain IgE antibodies. IgE binds to specific receptors on the surfaces of mast cells and basophils. When the sensitizing allergen cross-links the cell-bound particular IgE, the cells release or produce chemical mediators that cause the allergic symptoms [5]. Histamine that has already been generated is released by activated mast cells, along with freshly produced leukotrienes, prostaglandins, kinins, and other substances [6]. Due to vascular vasodilatation, vascular permeability, and increased mucus production, this mediator release ultimately causes an acute hypersensitive reaction with a scratchy, runny nose, and congestion. Both rhinorrhea and nasal congestion are brought on by the vascular leaking of plasma proteins [7]. After an allergen exposure, a late-phase response is brought on by the further release of agents of inflammation, which prolong nasal symptoms. During this late-phase response, cytokines and chemokines are released and produced, which attract more inflammatory cells. These cells then produce more agents of inflammation, which might aggravate nasal symptoms and get the nasal mucosa ready to respond more quickly and severely to subsequent allergen exposures. Atopic rhinitis affects up to 50% of asthma sufferers. The two primary vasoactive mediators are histamine and cysteinyl-leukotrienes released by eosinophils, T cells, mast cells, and basophils. These cells also regulate the production of IgE on a local and systemic level, interactions with the defense mechanism, and dialogue with the bone marrow [8]. Clinically, this causes rhinorrhea, sneezing, itching, and nasal obstruction, among other common symptoms. Inflammatory cells can invade other organs where molecules that serve as chemoattractants and adhesions are already present thanks to systemic circulation. As a result, in addition to causing Localized irritation, AR also causes generalized inflammation that may exacerbate inflammation in the upper and lower airways. As a result, there are several concomitant illnesses associated with AR, including problems sleeping, respiratory problems, sinusitis, nasal polyps, and serous otitis media [9].

Genetics

While allergic rhinitis is becoming increasingly common everywhere and there is evidence that the environment affects the disease, some people are more likely to be affected by a more severe form of allergic disease. Therefore, genetic research is crucial for comprehending disease pathogenesis. The symptoms of allergic rhinitis commonly begin in early infancy and peak after two, three, and four decades of age. However, it is not unusual for this condition to develop in childhood or later in adulthood. Similar to other atopic disorders, allergic rhinitis frequently affects numerous family members. However, the kind of atopic illness or specific sensitivities do not appear to be heritable as straightforward genetic traits. It is also suggested that particular allergen sensitivity and total IgE levels are influenced by genetic factors [9]. Growing research suggests that exposures throughout pregnancy and infancy have an impact on how allergy diseases develop. Through epigenetic processes, transient environmental stresses can permanently alter gene regulation and expression. Corticosteroid resistance and the extent of bronchial hyperresponsiveness in asthma have been linked to histone alterations. Dendritic cells and the development of human T-cells can both be modified by epigenetic processes [10]. According to research on mice, higher DNA methylation caused by a maternal diet high in methyl donors might increase the offspring's vulnerability to allergic inflammation [11]. As a result, the available data suggests that epigenetics plays a part in the onset and protracted nature of asthma and allergic rhinitis.

Early-phase reaction

Within minutes of allergen contact, sensitized people experience the early or immediate phase reaction, which lasts for around 2-3 hours. The degranulation of mast cells is one of the essential elements of the early phase response. Mast cells are common in the epithelial compartment of the nasal mucosa of the sensitized person and are quickly triggered following re-exposure to the allergens. Following the particular allergen's IgE that is specific to an allergen and is crosslinked around the outside of mastocytes, the early phase reaction is characterized by the degradation of mast cells and the release of a range of mediators that have already been created and those that have not yet been. Histamine, the primary trigger of allergic rhinitis, stimulates the sensory nerve terminals of the trigeminal branch of the Vth nerve, which causes sneezing. Additionally, rhinorrhea is caused by histamine activating the mucous glands, and nasal congestion is brought on by inflammatory mediators acting on the blood vessels [12].

Late-phase reaction

The late phase reaction, which happens 4-6 hours after antigen stimulation, often follows the early phase response. The symptoms of the late phase reaction, which remain for roughly 18 to 24 hours, include prolonged sneezing, rhinorrhea, and continuous nasal congestion. The late phase reaction is mostly inflammatory and is typified by an influx of inflammatory cells, such as basophils, eosinophils, and T lymphocytes. Mast cells generate and release many cytokines and chemokines, such as IL-4 and IL-13, which is crucial for the orchestration of the late phase response [13-15]. The late phase response is also thought to be mediated by a number of additional mediators, including the platelet-activating factor, major basic protein (MBP) and eosinophil cationic protein.

Symptoms and signs

AR symptoms include both non-nasal and rhino manifestations. Sneezing, nasal blockage, anterior or posterior rhinorrhea, and nose itch are examples of nasal symptoms [16]. These symptoms might last for several hours after an allergic response when allergens that induce mucosal inflammation are exposed [17]. As a result, the mucosa becomes more sensitive to the allergen that triggers an allergic reaction, additional allergens, as well as non-allergic triggers (e.g., strong scents and other irritants). Ocular symptoms include allergic rhinoconjunctivitis, which often affects AR patients and causes itching, redness, and tears of the eyes, which are known as non-nasal symptoms [18]. Postnasal drip, coughing, and itching of the palate are other symptoms. Among the disorders that might display hypersensitivity reactions include anaphylactic shock, bronchial asthma, allergic dermatitis, allergic conjunctivitis, and AR [19]. More than 30 percent of people with AR report devastating allergy symptoms that can seriously impede their ability to function and even fatal situations like anaphylaxis [20]. According to how long an AR patient has had their symptoms, the Allergic Rhinitis and Its Impact on Asthma (ARIA) recommendations further divide AR symptoms into intermittent and persistent categories. Intermittent symptoms occur on less than four days per week or for fewer than four consecutive weeks, whereas persistent symptoms last longer than four days per week or for longer than four consecutive weeks [21].

Treatment modalities in patients with allergic rhinitis

Anti-inflammatory treatments, symptomatic medicine, and allergy avoidance remain to be the mainstays of AR management. Intranasal antihistamines and new techniques for delivering intranasal steroids, which continue to be the basis of therapy for AR, are recent advancements in treatment.

Avoiding Allergens

In allergic rhinitis, it is important to know and find the trigger for the allergic reactions. Common allergens like dust mites are the most common indoor cause of allergic rhinitis. A clean and healthy environment should be maintained by the patient. Another common trigger is pollen, patients should cover their face and nose while traveling or in areas with more pollen.

Antihistamines

The most common first-line treatment for mild AR is antihistamines, although the initial generation of these drugs. Due to its numerous adverse effects on the neurological system, anticholinergic side effects, and cardiac toxicity, diphenhydramine and hydroxyzine are no anymore advised. As they exhibit improved effectiveness and safety profiles, newer generation antihistamines should be considered [22]. Olopatadine, levocabastine, and azelastine are examples of novel intranasal antihistamines that guarantee enhanced medication distribution of mediators exposure to allergic inflammation in the nasal mucosa in AR [23,24].

Leukotriene Receptor Antagonists

Then, leukotriene receptor antagonists, such as montelukast, zafirlukast, and pranlukast, inhibit the action of cysteinyl leukotrienes, a significant and strong mediator of allergies that causes allergic inflammation and a variety of allergy symptoms, including mucus production and nasal congestion. Leukotriene receptor antagonists were more effective than H1 antihistamines for symptoms throughout the night but not for symptoms during the day, according to meta-analysis research [25].

Nasal Decongestants

Through their agonistic activity at 1 and 2-adrenergic receptors on nasal mucosal endothelial cells, nasal decongestants alleviate nasal congestion symptoms by reducing mucosal edema [25]. Commonly used nasal sprays are oxymetazoline, phenylephrine, and pseudoephedrine. Nasal decongestants used excessively might result in nasal medicamentosa (i.e., a situation in which rebound blockage occurs after ceasing nasal decongestants) [26], which is treated by administering intranasal corticosteroid.

Corticosteroids

Intranasal corticosteroids are useful for treating both mild and moderate-severe AR in both children and adults by inhibiting the invasion of immune cells [25]. For severe or uncontrollable symptoms, systemic corticosteroids (by mouth or by injection) should only be used as a last resort.

Immunomodulating Therapies in Allergic Rhinitis

Treatment for AR that focuses on immune regulation aims to shift the normal courses of AR rather than bringing about a transformation to an immunologically ignorant or unresponsive state. Since some patients with AR do not benefit from standard medical care, allergen immunotherapy (AIT) is employed as a disease-modifying therapeutic approach [25]. There are two ways to administer AIT: subcutaneous immunotherapy (SCIT) or sublingual immunotherapy (SLIT). Targeting these indicators with AIT is promising since the early phase responses in AR are crucially mediated by basophils. The threshold for basophil activation can be lowered after one year of SLIT treatment for Parietaria, emphasizing the value of AIT in the management of illness and in slowing the course of the disease [26].

## Conclusions

An inflammatory infiltration consisting of several cell types is the defining feature of allergic rhinitis. IgE-antibodies that are specific for the allergen have developed and are attached to mast cells and other cell membranes, which is why these events only occur in subjects who have a history of allergy sensitivity. They don't happen in healthy persons since their nasal mucosa doesn't react to the same allergens in a noticeable way. The majority of asthma patients also have rhinitis, which is a significant independent risk factor for developing asthma. An intranasal glucocorticoid and nasal antihistamine combination may have cumulative effects. A sizable section of the population suffers from allergic rhinitis, which has a negative impact on their quality of life. The co-morbidities connected to rhinitis can further harm patients' quality of life. Identification and, if at all feasible, modification of underlying triggers are necessary for the treatment of rhinitis. The use of pharmacologic and nonpharmacologic therapy in a progressive manner may then be put into practice, usually with positive results for both patients and doctors. The mainstay of the therapy continues to be avoiding allergens. Comorbidities with AR place additional health and economic strain on individuals. Continuous use of topical nasal decongestants should be avoided to prevent rhinitis medicamentosa. Future research should focus on identifying further innovative pathways and therapeutic targets in the hopes of really helping people with allergic rhinitis.
